# Comparative analysis of dehazing algorithms on real-world hazy images

**DOI:** 10.1038/s41598-025-95510-z

**Published:** 2025-03-28

**Authors:** Chaobing Zheng, Wenjian Ying, Qingping Hu

**Affiliations:** 1 Institute of Robotics and Intelligent Systems, School of Information Science and Engineering, Wuhan University of Science and Technology, 947 Heping Avenue, Wuhan, 430081 China; 2 College of Weapon, Naval University of Engineering, Wuhan, 430033 China

**Keywords:** Energy science and technology, Engineering, Materials science, Mathematics and computing

## Abstract

Images captured in adverse weather conditions (haze, fog, smog, mist, etc.) often suffer significant degradation. Due to the scattering and absorption of these particles, various negative effects, such as reduced visibility, low contrast, and colour distortion are introduced into the image. These degraded images are unsuitable for many computer vision applications, including smart transportation, video surveillance, weather forecasting, and remote sensing. To ensure the reliable operation of such applications, a high-quality haze-free input image is essential, which is supplied by image dehazing techniques. This review categorises recent dehazing methods, highlighting popular approaches within each group. In recent years, deep learning methods and restoration-based techniques using priors have garnered attention, particularly for addressing challenges such as dense and non-homogeneous haze. In this paper, their typical candidates are compared by using real-world hazy images because most data-driven and neural augmentation methods are trained by using synthetic hazy images. Experimental results conducted on real-world hazy images reveal that physics-driven single-image dehazing algorithms exhibit a lack of robustness, while data-driven approaches perform well on thin hazy images but struggle in dense haze conditions. Neural augmentation algorithms, however, effectively combine the strengths of both approaches, offering a better overall solution. By identifying existing gaps in recent methods, this paper provides a valuable resource for both novice and experienced researchers, while pointing towards future directions in this rapidly advancing field.

## Introduction

Haze, an atmospheric phenomenon caused by the scattering of light by particles such as dust, smoke, and water droplets, severely degrades the quality of outdoor images. This degradation, manifesting as a loss of contrast and visibility, significantly impairs the performance of computer vision tasks such as object detection, tracking, and scene understanding. The impact of haze is especially critical in real-time applications, including autonomous driving, aerial surveillance, and environmental monitoring, where accurate visual information is crucial for decision-making. As a result, single image dehazing has become a prominent research area in the computer vision community, aimed at restoring clear images from hazy observations.

**Figure 1:**
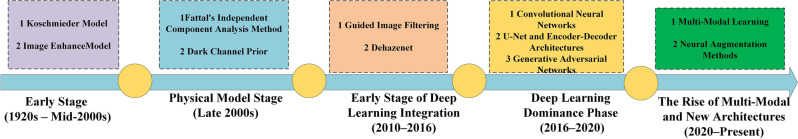
Different categories of image dehazing methods.

Single image dehazing is inherently an ill-posed problem, as the relationship between the hazy image and the corresponding clear image involves multiple unknown variables. Typically, the hazing process is modelled using the atmospheric scattering model, which relates the observed image intensity to the scene radiance, atmospheric light, and transmission map. Recovering the scene radiance (i.e., the clear image) from this model is challenging, especially when only a single hazy image is available. Over the past decade, various dehazing algorithms have been proposed to address this challenge, ranging from model-based methods to neural augmentation approaches. The detailed evolution of single image dehazing is illustrated in Fig. 1.

The model-based approaches are grounded in the physical principles of light scattering, which provide a solid theoretical foundation for the dehazing process. The underlying atmospheric scattering model offers a comprehensive understanding of how haze affects images, which allows for a structured approach to address the problem of haze removal. This approach can, in principle, recover a significant amount of detail, especially when the transmission map and atmospheric light are accurately estimated.

While the model-based dehazing methods have clear advantages, it also suffer from several limitations, particularly in handling complex real-world scenarios. These challenges arise primarily from the assumptions and simplifications inherent in the physical model, which do not always hold true in practice.

In recent years, deep learning has emerged as the dominant paradigm in single image dehazing^1–4^. Data-driven methods have achieved remarkable success by learning complex representations from large datasets. These models can effectively capture the intricate relationship between hazy and clear images, producing high-quality dehazing results. Moreover, advanced techniques such as GANs and transformers have further pushed the boundaries of dehazing performance, offering better generalisation and visual realism.

Data-driven methods are highly adaptive and can be trained on large datasets to learn the specific characteristics of various environmental conditions. This adaptability is particularly advantageous when dealing with real-world images that may not strictly adhere to the assumptions of the physical models. As such, deep learning methods can handle a wider variety of haze levels, from light mist to dense fog, as well as more complex atmospheric conditions that may not be easily modeled by traditional algorithms.highly adaptive and can be trained on large datasets to learn the specific characteristics of various environmental conditions. This adaptability is particularly advantageous when dealing with real-world images that may not strictly adhere to the assumptions of the physical models. As such, deep learning methods can handle a wider variety of haze levels, from light mist to dense fog, as well as more complex atmospheric conditions that may not be easily modeled by traditional algorithms.

A key limitation of deep learning-based dehazing algorithms is their reliance on large, annotated training datasets. The performance of these models heavily depends on the quality and quantity of the training data, which can be a significant challenge when trying to cover the vast diversity of possible real-world scenarios. In some cases, insufficient or biased training data can lead to overfitting, resulting in poor generalization to unseen data.

Since it is very difficult or even impossible to capture a hazy image and its corresponding hazy-free image simultaneously, almost all data-driven dehzaing algorithms are trained by using synthetic hazy images. Even though these algorithms work well in synthetic hazy images, a natural question is “do they also perform well on real-world hazy images?”. The main objective of this paper is to provide an answer to this question. Besides the data-driven methods, there are also physics-driven methods and neural augmentation methods. These two types are also tested by using the data-set presented in^5^. Experimental results demonstrate that physics-driven dehazing algorithms perform well on real-world hazy images, while data-driven ones are able to process thin hazy images but perform poorly in dense hazy conditions. Neural augmentation algorithms effectively combine the strengths of both types of approaches, providing a better solution. It aims to provide researchers with a thorough guide to the current landscape of image dehazing, helping to identify directions for future work.

## Haze imaging model

**Figure 2:**
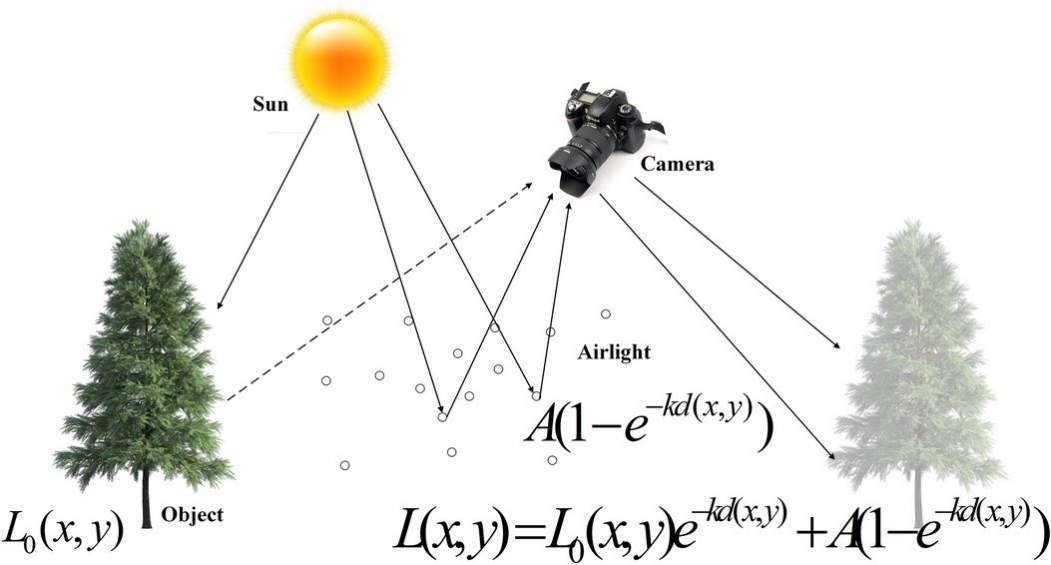
Haze imaging model.

The haze imaging model was first introduced by Koschmieder^6^ and later refined by McCartney^7^, as shown in Fig. 2. It is expressed as follows:

1 $$\begin{aligned} L(x,y) = L_0(x,y) e^{-kd(x,y)} + A(1-e^{-kd(x,y)}) \end{aligned}$$

where *L*(*x*, *y*) is the captured hazy image, $L_0(x,y)$ denotes the haze-free image, *k* is the atmospheric scattering coefficient, *d*(*x*, *y*) represents the scene depth; $e^{-kd(x,y)}$ is the transmission map; and *A* is the atmospheric light.

Single image dehazing poses a significant challenge because the transmission map *t* depends on unknown and varying scene depth. Two main categories of algorithms have emerged: model-based and data-driven approaches. Many model-based methods rely on the haze formation model (1), where both the atmospheric light *A* and the transmission map *t* must be estimated. Traditional methods for estimating atmospheric light, such as those proposed in^8,9^, are derived from this model. In these approaches, *A* is typically assumed to be constant, though it can be refined using a data-driven strategy, such as the neural augmentation proposed in this work. Since the number of unknown variables exceeds the available observations, single image dehazing is inherently an ill-posed problem.

**Figure 3:**
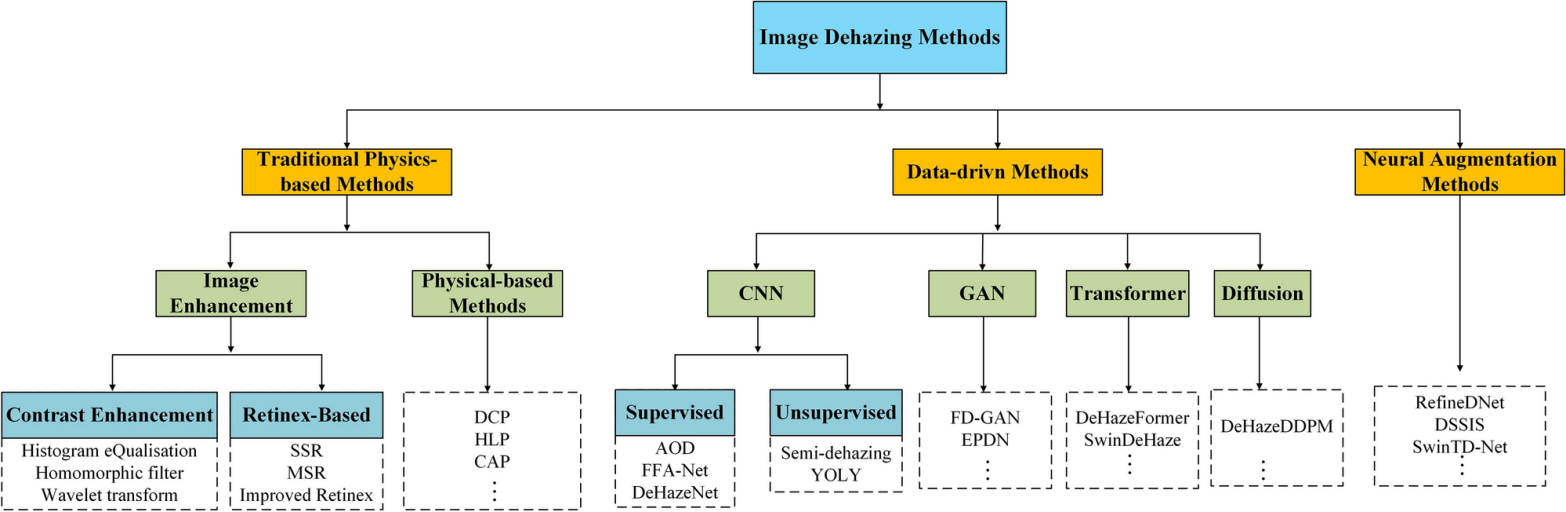
Different categories of image dehazing methods.

Based on the approaches for solving this ill-posed problem, single image dehazing algorithms can be categorised into the following three types: traditional physics-based methods, data-driven methods, and neural augmentation-based methods, as shown in Fig. 3. The following sections will provide a detailed introduction to each of these approaches.

## Model-based methods

Traditional model-based image dehazing algorithms are mainly divided into two categories: image enhancement-based methods and physical-based methods.

### Image enhancement-based methods

Image enhancement-based dehazing algorithms enhance image details and improve contrast, making the image appear clearer. These methods have a broad range of applicability. Notable algorithms in this category include Retinex algorithms, histogram equalisation, partial differential equations (PDE)-based methods, and wavelet transform algorithms. The Retinex algorithm enhances image quality by eliminating the effects of reflectance components based on imaging principles. Histogram equalisation improves the distribution of pixel intensities, thereby amplifying image details. PDE-based methods treat the image as a partial differential equation and enhance contrast by calculating the gradient field. Wavelet transform algorithms decompose the image, magnifying the useful components. Numerous improved algorithms based on the principles of image enhancement have since been developed^10–14^.

### Physical-based methods

Physical-based dehazing algorithms, on the other hand, rely on assumptions about the statistical properties of clean images and the way haze affects these properties. By observing and analysing large sets of hazy and haze-free images, these methods identify the mapping relationships between them. Using this information, they reverse the image formation process of hazy images to recover a clear image^13–17^. For instance, the Dark Channel Prior, which assumes that in most non-sky regions, at least one colour channel of many pixels has very low intensity, remains one of the most influential works in this domain. Despite its success, DCP is known to perform poorly in scenes with bright objects, such as snow or white buildings, and in situations where the haze^8^ is particularly dense. A novel haze line prior (HLP) was introduced in^15^ to address this challenge. The HLP is based on the observation that the colours in a haze-free image can typically be represented by a finite number of distinct clusters in RGB space^16^. In hazy images, pixels within each cluster form a haze line, and the HLP assumes that each haze line contains at least one haze-free pixel. An initial transmission map is estimated using the HLP, and the resulting artifacts are reduced through a weighted least squares (WLS) framework^17–19^. While the priors in^8^ and^15^ are robust across varying haze levels, both the dark channel prior (DCP) in^8^ and the HLP in^15^ fail to hold for pixels in sky regions. A dark direct attenuation prior (DDAP) is introduced in^5^ to tackle these two inherent challenges. Initially, a transmission map is derived using DDAP. Instead of applying edge-preserving smoothing filters as done in^8,20,21^, the morphological artifacts introduced by DDAP are mitigated through a novel haze line averaging algorithm. This algorithm first estimates the distance of each pixel from the origin in the haze-free image using the initial transmission map, then averages the values of pixels along the same haze line to refine the estimate. Finally, the refined pyramid is collapsed to produce the dehazed image. Ju et al.^22^ found that when an image is divided into *n* regions with similar scene depth, the brightness of both hazy and clear images are positively correlated with scene depth, referred to as the region-line prior. By leveraging this prior along with the atmospheric scattering model, dehazing can yield ideal results across various levels of haze. However, real-world scenarios are more complex, with uneven distributions and random noise, which may violate the prior assumptions and lead to algorithmic failure. The traditional atmospheric scattering model fails to account for the noise term, which can lead to the amplification of noise during the dehazing process. To address this limitation, Zhou et al.^23^ incorporated the noise term into the atmospheric scattering model. Subsequently, an alternating optimisation approach was employed to solve the proposed model, effectively achieving simultaneous haze removal and noise suppression.

Traditional dehazing methods, particularly those based on physical models like the atmospheric scattering model, are relatively straightforward and interpretable. They directly employ well-established physical principles, making their operation clear and their implementation simpler. These traditional methods often have lower computational demands, as they do not require extensive training on large datasets. This characteristic can lead to significant time and resource savings, making such methods more suitable for real-time applications. However, their reliance on fixed models and parameters can result in inconsistent performance across different types of images and varying environmental conditions, leading to variability in the quality of the dehazed results.

## Data-driven methods

Data-driven single image dehazing algorithms present data-driven solutions that effectively address the limitations of traditional handcrafted priors. These methods have demonstrated exceptional performance and can be broadly classified into four key categories: Convolutional Neural Networks (CNNs), Generative Adversarial Networks (GANs), Transformers, and Diffusion models. Each of these approaches has made substantial contributions to advancing the state-of-the-art in image dehazing, offering unique advantages in tackling the complexities inherent in dehazing tasks. The subsequent sections provide a comprehensive analysis of each category, highlighting their innovations and contributions to the field.

### Convolutional neural networks (CNN)-based methods

Deep learning, particularly Convolutional Neural Networks (CNNs), has revolutionised single image dehazing. CNN-based methods learn hierarchical feature representations from large datasets, enabling them to capture the complex patterns of haze and recover clear images with high fidelity. In contrast to prior-based methods, CNNs do not rely on explicit physical models or handcrafted features, allowing them to generalise better to a wide variety of scenes. Based on different learning strategies, CNN-based dehazing methods can be further subdivided into supervised and unsupervised approaches. Supervised methods rely on large annotated datasets of paired hazy and clear images for training, ensuring accurate dehazing performance through direct supervision. These models typically achieve high-quality results but require extensive labelled data, which can be difficult to obtain in real-world scenarios. In contrast, unsupervised methods aim to overcome this limitation by learning from unpaired or unlabelled data. These approaches often leverage generative models or domain adaptation techniques to infer clear images from hazy inputs without the need for paired training data, offering greater flexibility and applicability in diverse and challenging environments. Both learning paradigms have significantly advanced the field, with each offering distinct advantages depending on the availability of labelled data and the specific application requirements.

*supervised methods* In recent years, several influential supervised CNN-based dehazing models have emerged, significantly advancing the field of image dehazing^24,24–27^. One of the pioneering models, DehazeNet^28^, introduced a deep convolutional network specifically designed to estimate the transmission map, which is subsequently employed to reconstruct the haze-free image. This model was among the first to successfully harness the capabilities of CNNs for the purpose of image dehazing, achieving impressive results across a variety of datasets, thereby establishing a benchmark in the field. However, despite its effectiveness, DehazeNet fundamentally relies on the traditional atmospheric scattering model as its underlying framework. This dependence limits its adaptability and performance in more complex or non-homogeneous haze scenarios, where the assumptions inherent in the scattering model may not hold true. Consequently, while DehazeNet marked a significant advancement in dehazing techniques, its reliance on fixed physical models underscored the necessity for more flexible and generalisable approaches that can effectively accommodate diverse environmental conditions and the complexities of real-world scenes. This recognition has paved the way for subsequent research aimed at developing more robust and adaptable dehazing methods that can transcend the limitations of earlier models.

A significant breakthrough in CNN-based dehazing occurred with the introduction of AOD-Net^29^, which streamlined the dehazing process by directly generating the dehazed image, bypassing the need to estimate intermediate variables such as the transmission map or atmospheric light. This end-to-end framework marked a departure from traditional methods, offering a more efficient and simplified approach to dehazing. By eliminating the dependency on intermediate computations, AOD-Net not only enhanced dehazing performance but also significantly improved computational efficiency, making it highly suitable for real-time applications. The model?s ability to directly predict a clear image without relying on atmospheric priors demonstrated a more flexible and robust solution for various haze conditions, establishing a new direction for future research in image dehazing.

Multi-scale networks have garnered significant attention in recent dehazing research due to their ability to capture and process image features at different levels of detail^5,30–32^. One notable example is the Multi-Scale Boosted Dehazing Network (MSBDN)^33^, which employs a multi-scale feature fusion strategy to enhance the preservation of fine details in dehazed images. By learning and integrating features across multiple scales, MSBDN demonstrates the ability to effectively address both fine-grained textures and large-scale structures in hazy images, ensuring comprehensive restoration. This approach has achieved state-of-the-art performance on several widely used benchmarks, demonstrating its efficacy in challenging dehazing tasks. However, the method?s computational complexity poses a limitation, making its practical deployment, especially in real-time applications, more challenging.

*Unsupervised methods* Due to the difficulty of obtaining paired dehazing datasets in real-world scenarios, there has been a growing interest in unsupervised CNN algorithms^34–39^. These approaches do not require matched hazy and haze-free image pairs for training, making them particularly valuable in practical applications where such data is scarce or unavailable. Unsupervised methods leverage alternative strategies, such as self-supervision or the use of unlabelled data, to learn effective dehazing representations. By circumventing the need for paired data, these algorithms can enhance their applicability across a wider range of conditions, ultimately contributing to more robust and adaptable image dehazing solutions. This shift towards unsupervised learning techniques represents a significant advancement in the field, enabling researchers to explore novel methodologies that can operate effectively under the constraints of real-world data availability.

Li et al. propose semi-supervised architecture that integrates both supervised and unsupervised learning branches to enhance image dehazing performance^38^. The supervised branch employs various loss functions to guide the network in generating high-quality dehazed images. Conversely, the unsupervised branch leverages the inherent properties of clear images, specifically focusing on the sparsity of the dark channel and gradient priors, to impose additional constraints on the network’s learning process. This dual-branch approach allows the model to benefit from both labelled synthetic datasets and unlabelled real-world images, facilitating training in an end-to-end manner. By combining these methodologies, the proposed network aims to achieve robust dehazing capabilities across diverse image conditions.

YOLY is proposed in^37^, which utilises a novel architecture comprising three interconnected subnetworks designed to decompose the observed hazy image into distinct latent layers: the scene radiance layer, the transmission map layer, and the atmospheric light layer. This decomposition process allows for a more nuanced understanding of the image components contributing to the haze. Following this separation, the three layers are recombined to reconstruct the hazy image in a self-supervised fashion, facilitating an effective learning mechanism. This approach not only enhances the model’s ability to recover clearer images but also improves the interpretability of the underlying dehazing process. By leveraging self-supervision, YOLY optimises its performance without relying heavily on extensive labelled datasets, thus making it a robust solution in the realm of image dehazing.

Unsupervised deep learning approaches for single image dehazing present innovative methodologies to circumvent the limitations of traditional supervised models. While these algorithms offer advantages such as reduced dependency on labelled datasets and enhanced adaptability, they also face challenges related to model generalisation and complexity.

### Generative adversarial networks (GANs)

Generative adversarial networks (GANs) have gained traction in image dehazing due to their ability to generate realistic and visually pleasing results^40–46^. GANs consist of two networks: a generator that produces dehazed images and a discriminator that distinguishes between real and generated images. The adversarial training process forces the generator to produce more accurate dehazed images.

Zhang et al. incorporates a pyramid structure to extract both global and local haze features^40^, enabling it to handle dense and spatially varying haze. By combining pyramid levels with dense connections, this model captures complex haze patterns and achieves state-of-the-art performance on several benchmark datasets. However, GAN-based models can sometimes introduce artefacts, particularly in challenging lighting conditions.

CycleGAN has been adapted for image dehazing^41^, offering the advantage of training without the need for paired hazy-clear image datasets, which are often challenging to acquire. This is achieved by employing a cycle-consistency loss, which ensures that the generated dehazed images remain faithful to the original scene by reconstructing the hazy images from the dehazed output, thereby enforcing consistency. While this unsupervised learning approach provides greater flexibility and circumvents the dependency on paired datasets, unsupervised GAN-based models, such as CycleGAN, tend to fall short compared to supervised methods, particularly in the restoration of fine details. This performance gap highlights the trade-off between data availability and output quality in dehazing tasks using unsupervised techniques.

### Transformer-based methods

Transformers, initially introduced in natural language processing, have recently been adopted for single image dehazing due to their ability to capture long-range dependencies and global context within an image. Unlike traditional convolutional networks, which focus on local features through limited receptive fields, transformers excel at modeling global relationships between pixels, making them particularly suited for dehazing tasks where both local textures and global structures are crucial^47–52^. Several transformer-based approaches have been developed for image dehazing, leveraging self-attention mechanisms to effectively manage the complex scattering of light caused by haze. These models are able to process entire images holistically, capturing fine details as well as large-scale scene structures. A key advantage of transformers lies in their capacity to model the non-uniform nature of haze across an image, allowing for more precise restoration.

The Image Processing Transformer (IPT) applies the transformer architecture to image dehazing and other low-level vision tasks^52^. By utilising self-attention, IPT can effectively capture both local and global features, leading to highly accurate dehazing results. However, transformer models are computationally expensive, requiring large amounts of memory and processing power, which limits their real-world applicability. Building on the observation that several key design elements of the Swin Transformer^53^ are suboptimal for image dehazing, Song et al. proposed DehazeFormer^47^, a tailored variant designed specifically to address the challenges of this task. They introduced modifications to crucial components such as the normalization layer, activation function, and spatial information aggregation scheme to better align the model?s capabilities with the demands of dehazing.These architectural adjustments enable DehazeFormer to outperform conventional transformer-based methods in the specific domain of image dehazing, addressing both the global and local challenges presented by varying haze densities and non-uniform atmospheric conditions. Zheng et al.^54^ proposed a Transformer-guided CycleGAN framework (Dehaze-TGGAN) that incorporates an additional attention mechanism from the frequency domain. Experimental results on both simulated and measured optical remote sensing data demonstrate significant improvements in recognition accuracy and computational efficiency. However, the Transformer-based dehazing algorithms suffer from high computational complexity and increased memory usage. Additionally, the results from the GAN-based approach often exhibit exaggerated colour saturation, making them appear unnatural.

However, while transformer-based models have demonstrated competitive performance, they come with increased computational demands and memory requirements, limiting their deployment in real-time or resource-constrained environments. Recent efforts have aimed at optimizing transformer architectures to balance computational efficiency with dehazing accuracy, such as through hybrid approaches that combine convolutional layers with transformer modules to harness the strengths of both architectures.

### Diffusion methods

Diffusion models have recently emerged as a promising approach in single image dehazing^55–60^, leveraging their generative capabilities to tackle the complexities of haze removal. Diffusion models operate by gradually denoising a noisy image, guided by a probabilistic model, to progressively reconstruct the desired clean image. This iterative refinement process allows the model to recover lost details and enhance image clarity in challenging environments.

Yu et al. proposes a DDPM-based image dehazing framework (DehazeDDPM)^59^, which operates in two stages. First, the dehazing process is modelled using the Atmospheric Scattering Model (ASM) to adjust the data distribution, aligning it more closely with clear images and improving fog-awareness. In the second stage, the generative power of DDPM compensates for the significant information loss induced by haze, effectively restoring the image.

However, despite their powerful generative capacity, diffusion models face certain challenges in practical applications. One key issue is the computational cost associated with the iterative denoising process, which typically requires a large number of inference steps to achieve high-quality results. This can make real-time or resource-constrained deployments difficult. To address this, recent research has focused on reducing the number of diffusion steps without compromising output quality, aiming to balance the trade-off between performance and efficiency.

## Neural augmentation methods

Neural augmentation has recently emerged as an innovative technique in the realm of image processing, particularly for challenging tasks like single image dehazing^61–64^, High Dynamic Range Imaging^65–67^. Neural augmentation refers to the enhancement of model performance through the integration of additional neural network modules.

Zhao et al. propose a two-stage weakly supervised dehazing framework, RefineDNet^62^. In the first stage, RefineDNet utilises the dark channel prior to enhance visibility. The second stage refines the initial dehazing results through adversarial learning with unpaired foggy and clear images, improving the realism of the output. RefineDNet effectively combines the strengths of prior-based and learning-based approaches. Li et al. proposed a novel dehazing algorithm based on neural augmentation^[[61^. Initially, the transmission map and atmospheric light are estimated using model-based methods, followed by refinement through a dual-scale generative adversarial network (GAN). This neural augmentation approach achieves fast convergence, addressing issues where purely data-driven methods may struggle to converge efficiently. Zhou et al.^68^ proposed a physics prior-guided deep image dehazing model, SwinTD-Net, which is trained using both supervised and self-supervised learning strategies. This model integrates the advantages of physical priors and the self-attention mechanism inherent to Transformers, enabling the network to perform feature learning under the guidance of physical priors. This approach enhances the model’s generalisation capability, allowing it to achieve effective restoration on both synthetically generated hazy images and real-world hazy images. Ju et al.^69^ discovered that the normalized DCT coefficients of high-quality images almost overlap with those of low-quality images within the same interval. Leveraging this DCT prior knowledge, they applied it for image enhancement, achieving ideal results across various image enhancement tasks. This “all-in-one” image enhancement model has become a prevailing trend^70^.

Neural augmentation-based dehazing algorithms combine the strengths of both traditional physics-based methods and deep learning-based methods, while also mitigating the limitations inherent to each. By leveraging this hybrid approach, these algorithms reduce dependency on large datasets, enhancing their adaptability and robustness. This fusion of methodologies not only preserves the interpretability and efficiency of model-driven techniques but also benefits from the superior performance and flexibility offered by data-driven methods. Consequently, neural augmentation provides a more resilient and effective solution for image dehazing across diverse conditions.

In addition to the aforementioned methods based purely on visible light imaging for dehazing, infrared and visible light image fusion has also emerged as an effective solution for removing dense fog^71^, yielding promising results. By leveraging the complementary information provided by both infrared and visible light images, this approach can enhance the visibility and clarity of foggy scenes, as infrared images can capture details obscured by fog that are invisible to conventional visible light imaging. This fusion technique has shown significant improvements in both dehazing performance and the preservation of image details, making it a valuable alternative in challenging foggy conditions.

## Experiment results and analysis

### Dataset

With the increasing emphasis on deep learning-based image dehazing, relevant dehazing datasets are continuously being introduced, thereby accelerating progress in this field. Table 1 provides an overview of the eight most commonly used dehazing datasets. As shown in Table 1, aside from the “RESIDE” (Realistic Single Image Dehazing)^72^ and “MDID” (Multi-Degraded Image Dataset) datasets^73^, most others are relatively limited in size. For instance, the “HazeRD” dataset^74^ contains just 15 clear images and 75 synthetically generated hazy images, each exhibiting varying levels of haze. On the other hand, the “I-HAZE” dataset^75^ comprises 35 pairs of indoor images, each including both hazy and corresponding haze-free versions. These foggy images were produced under real-world overcast conditions using a dedicated haze machine. In comparison, the “O-HAZE” dataset^76^ offers 45 pairs of hazy and clear images captured in outdoor environments. Similarly, both the “Dense-HAZE”^77^ and “NH-HAZE”^78^ datasets each consist of 55 pairs of hazy and clear outdoor images, primarily characterized by grayish haze.

**Table 1:** Dehazing datasets.

Dataset	Year	Number	Type
HazeRD^74^	2017	75	Model synthesis
I-HAZE^75^	2018	35	Model synthesis
O-HAZE^76^	2018	45	Model synthesis
Dense-HAZE^77^	2019	55	Model synthesis
NH-HAZE^78^	2020	55	Model synthesis
RESIDE^72^	2019	87,125	Model synthesis and Real
MDID^73^	2020	30,346	Model synthesis
BeDDE^79^	2020	208	Real
DSSID^61^	2022	79	Real

### Evaluation metrics

Two primary methods are commonly employed to evaluate the quality of single-image dehazing: automated evaluation metrics and subjective assessment through the Human Visual System (HVS). For automated evaluation, a suite of metrics is typically used, with five of them being specifically designed for assessing image dehazing performance. These include Peak Signal-to-Noise Ratio (PSNR), Mean Squared Error (MSE), Structural Similarity Index (SSIM)^80^, BRISQUE^81^, NIQE^82^, and MetaIQA^83^. PSNR and SSIM are the most widely adopted full-reference metrics, providing an objective comparison of the dehazed image with a reference, typically the ground truth. In addition to these commonly used metrics, several other specialized measures are designed specifically for image dehazing, such as Visibility Index (VI)^79^, Retained Information (RI)^79^, and Dehazing Quality Index (DHQI)^84^, which are particularly focused on assessing the perceptual improvements and the retention of scene details in the dehazed output. These evaluation criteria collectively contribute to a more comprehensive and nuanced understanding of the effectiveness of dehazing algorithms, accounting for both objective measures of image quality and perceptual characteristics as evaluated by the human observer.

To rigorously evaluate and analyse the robustness of the various dehazing algorithms, we conducted comparative experiments using real-world hazy images from the dataset presented in^61^. In addition, to ensure an objective performance comparison across different algorithms, we employed DHQI in^84^ and FADE in^85^ , which utilise no-reference dehazing metrics. These metrics are particularly suitable as they do not require corresponding ground-truth images, making them ideal for assessing dehazing performance in real-world scenarios where reference images are typically unavailable. The use of these established benchmarks enhances the reliability of the evaluation, offering a comprehensive and unbiased assessment of the algorithms’ effectiveness.

### Comparison of the state of the art methods based on dehazing assessment

We compare fifteen state-of-the-art dehazing algorithms drawn from three distinct categories: traditional physics-based methods DCP^8^, HLP^15^ and DDAP^5^, deep learning-based methods MSBDN^33^, FFA-Net^25^, DeHazeNet^28^, PSD^26^, MSFNet^27^, SCANet^24^, YOLY^37^, FD-GAN^45^, EPDN^46^, DehazeFormer^47^, and neural augmentation-based methods RefineDNet^62^, DSSID^61^. To ensure the fairness and integrity of the experiments, all data-driven models were implemented using their official pre-trained versions, thus avoiding potential inconsistencies that may arise from re-training. This approach guarantees that the results remain unbiased and directly comparable, providing a reliable evaluation of the performance of these methods. The following sections will offer a comprehensive analysis of the experimental results and draw insights into the strengths and limitations of each category of dehazing algorithms.

**Figure 4:**
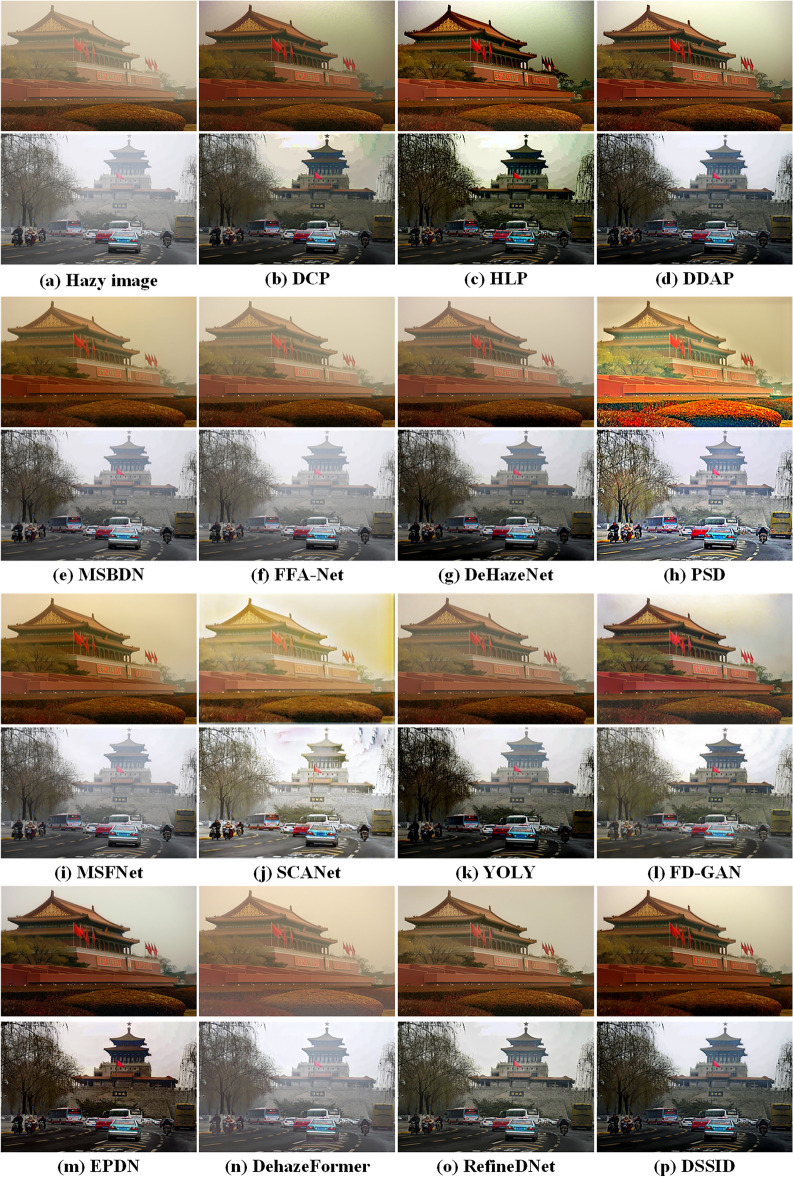
Comparison of different haze removal algorithms on thin hazing images. From left to right, hazy images, dehazed images by DCP^8^, HLP^15^, DDAP^5^, MSBDN^33^, FFA-Net^25^, DeHazeNet^28^, PSD^26^, MSFNet^27^, SCANet^24^, YOLY^37^, FD-GAN^45^, EPDN^46^, DehazeFormer^47^, RefineDNet^62^, DSSID^61^, respectively.

**Figure 5:**
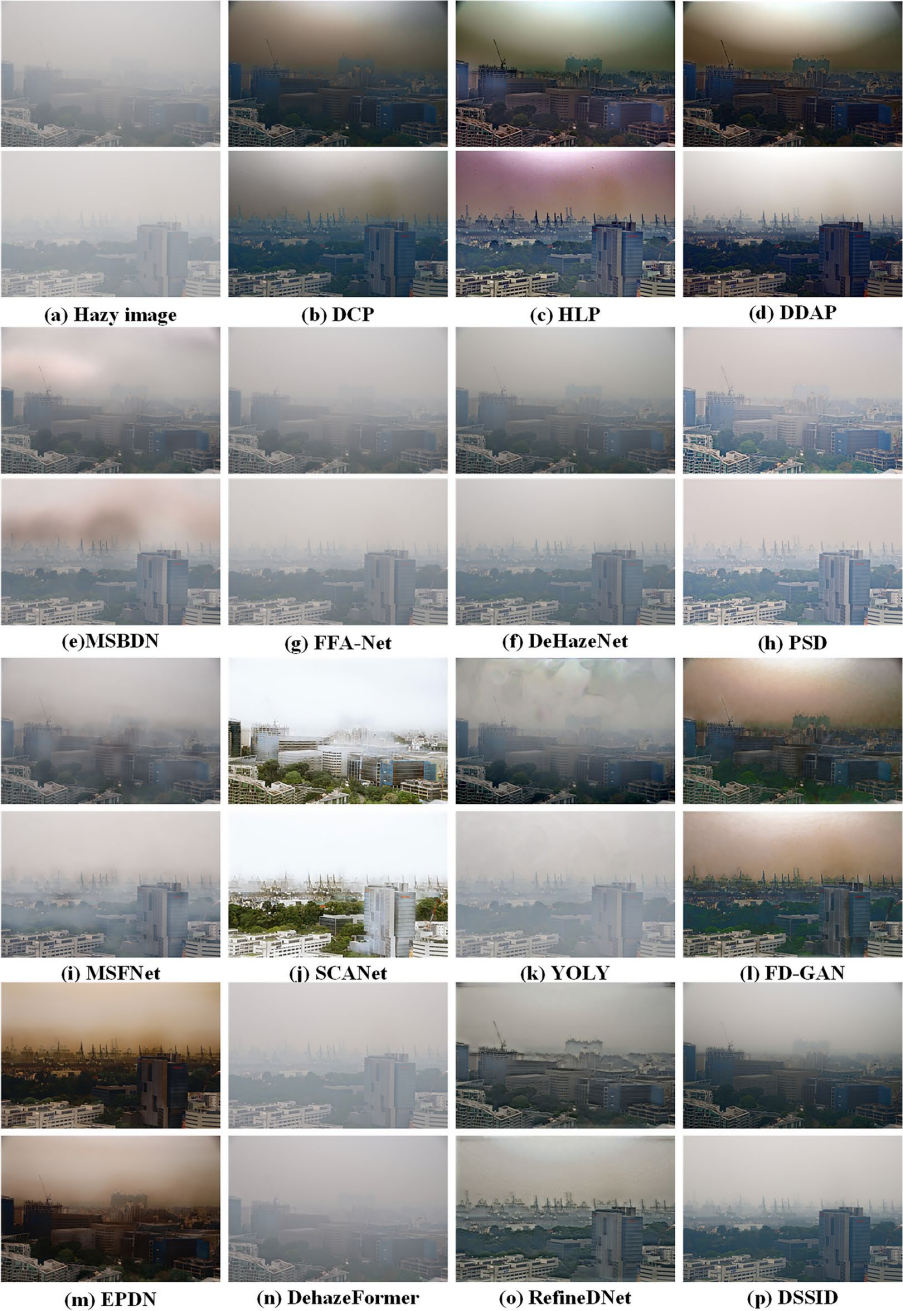
More comparison of different haze removal algorithms on dense hazing images. From left to right, hazy images, dehazed images by DCP^8^, HLP^15^, DDAP^5^, MSBDN^33^, FFA-Net^25^, DeHazeNet^28^, PSD^26^, MSFNet^27^, SCANet^24^, YOLY^37^, FD-GAN^45^, EPDN^46^, DehazeFormer^47^, RefineDNet^62^, DSSID^61^, respectively.

All fifteen algorithms are first evaluated from a subjective perspective, focusing on three key aspects: noise in sky or dense hazing regions, preservation of details, and retention of colour information. As shown in Figs. 4 and 5, Traditional physics-based methods DCP^8^ and HLP^15^ are both effective at removing haze and enhancing detail information. However, algorithm DCP^8^ significantly amplifies noise in the sky region, while algorithm HLP^15^ introduces noticeable halo artifacts in the sky and exhibits colour distortion. In contrast, DDAP^5^ demonstrates better noise suppression in the sky region and maintains superior colour fidelity.

Among the data-driven algorithms, MSFNet^27^ and MSBDN^33^ adopts a multi-scale strategy that enhances detail information in thin hazy images, yet its performance deteriorates under dense hazy conditions. DeHazeNet^28^, YOLY^37^, and PSD^26^ are all based on learning the atmospheric scattering model, demonstrating a certain degree of dehazing capability and improving detail preservation in thin hazy images. However, their effectiveness diminishes significantly in dense haze in Fig. 5.

FD-GAN^45^ and EPDN^46^ are GAN-based dehazing algorithms, designed in an end-to-end manner. Compared to other end-to-end CNN-based algorithms such as FFA-Net^25^ and DehazeFormer^47^, they significantly enhance detail preservation. However, both FD-GAN^45^ and EPDN^46^ suffer from noticeable colour distortions. In contrast, FFA-Net^25^ and DehazeFormer^47^, while less prone to colour artifacts, show poor performance on real-world hazy images due to their strong dependence on the training dataset. Similarly, the end-to-end CNN-based method SCANet^24^, while showing enhanced detail preservation when applied to a different domain, exhibits significant colour distortion, particularly in the sky regions. This issue arises due to its strong reliance on the training dataset, which limits its generalisation across different domains. These algorithms are all data-driven dehazing methods. While they perform well in thin hazy conditions, their effectiveness is significantly reduced in the presence of dense haze. RefineDNet^62^ and DSSID^61^ are both based on neural augmentation methods and operate on the atmospheric scattering model, demonstrating commendable dehazing performance. Compared to the other dehazing algorithms mentioned above, both RefineDNet^62^ and DSSID^61^ can achieve better results in both thin and dense hazy conditions. However, RefineDNet^62^ produces halo effect in dense fog, as shown in Fig. 5.

Since the ground-truth images do not exit, all the fifteen algorithms are also compared from the DHQI and FADE point of view as in the algorithms in^84^ and^85^. The average DHQI and FADE values of the 79 real-world outdoor hazy images are given in Table 2 and 3. Detail-enhanced images can achieve higher DHQI scores and lower FADE values.

**Table 2:** Average DHQI ($\uparrow$) values of 79 real-world outdoor hazy images for different algorithms.

DCP^8^	HLP ^15^	DDAP^5^	MSBDN^33^	FFA-Net^25^
51.92	52.75	60.97	54.32	55.33
DeHazeNet^28^	PSD^26^	MSFNet^27^	SCANet^24^	YOLY^37^
51.92	52.75	54.32	42.29	51.00
FD-GAN^45^	EPDN^46^	DehazeFormer^47^	RefineDNet^62^	DSSID^61^
51.00	63.80	50.60	57.57	62.58

**Table 3:** Average FADE ($\downarrow$) values of 79 real-world outdoor hazy images for different algorithms.

DCP^8^	HLP^15^	DDAP^5^	MSBDN^33^	FFA-Net^25^
0.6771	0.3980	0.4600	1.3745	1.8289
DeHazeNet^28^	PSD^26^	MSFNet^27^	SCANet^24^	YOLY^37^
0.9953	0.6679	1.4394	0.7323	0.7721
FD-GAN^45^	EPDN^46^	DehazeFormer^47^	RefineDNet^62^	DSSID^61^
0.6261	0.5312	50.60	0.6690	0.5883

## Conclusion remarks and discussions

Image dehazing algorithms primarily fall into three categories: traditional model-based methods, data-driven approaches, and neural augmentation techniques. Data-driven single image dehazing algorithms can achieve effective results in thin hazing images, yet their performance significantly deteriorates in dense hazing images. Model-driven single image dehazing algorithms, on the other hand, suffer from a lack of robustness. Neural augmentation algorithms integrate the advantages of both approaches, providing a more comprehensive solution. The aforementioned algorithms yield satisfactory results on simulated datasets, particularly for thin haze images. However, their performance deteriorates on dense haze images, indicating that single image dehazing remains an area for further research. We will explore several potential research directions in the following sections.

The “domain gap” problem in image dehazing arises when training samples are typically generated through simulation, leading to a significant disparity between simulated and real-world images. As a result, models trained on simulated data often perform poorly or fail when applied to real-world scenarios. Additionally, employing non-paired samples in a semi-supervised learning approach introduces challenges such as training instability and inconsistent outputs. Addressing this domain gap is critical and represents a key area for future research in image dehazing.

Existing methods focus on single image haze removal for human perception. It is also highly demanded to study single image dehazing for machine perception.

## Data Availability

The datasets used and/or analyzed during the current study are available from the corresponding author on reasonable request. The used dataset is publicly available; you can access it at :https://github.com/zhengchaobing/Multi-scale-Single-Image-Dehazing-Using-Laplacian-and-Gaussian-Pyramids.
